# Effect of multimodal home-based prehabilitation on objectively measured physical activity in patients undergoing elective cardiac or non-cardiac major surgery: secondary outcomes from a randomised controlled trial

**DOI:** 10.1186/s13741-025-00554-4

**Published:** 2025-07-04

**Authors:** Thomas Vetsch, Simone Wen-Shi Dueblin, Prisca Eser, Christian M. Beilstein, Patrick Y. Wuethrich, Matthias Wilhelm, Dominque Engel

**Affiliations:** 1 Department of Anaesthesiology and Pain Medicine, Inselspital, Bern University Hospital, University of Bern, Bern, CH-3010 Switzerland; 2 Centre for Rehabilitation & Sports Medicine, Inselspital, Bern University Hospital, University of Bern, Bern, Switzerland; 3 Graduate School for Health Sciences, University of Bern, Bern, Switzerland

**Keywords:** Prehabilitation, Physical activity, Accelerometer, Telemedicine

## Abstract

**Objective:**

To assess physical activity (PA) measured in steps per day in the preoperative period in high-risk cardiac and non-cardiac surgical patients receiving home-based tele-supervised prehabilitation compared to standard of care and to compare steps per day with raw acceleration metrics.

**Study design:**

It is an analysis of secondary outcome data of a prospective, two-arm parallel group, randomised controlled trial.

**Setting:**

It is a single university hospital in Switzerland.

**Participants:**

These are patients ≥ 65 years awaiting elective cardiac or non-cardiac major surgery with a proven fitness deficit measured by a cardiopulmonary exercise test (CPET). Analysis of PA data after successfully enrolling 200 patients (167 with complete data) in the trial. Average age was 73.8 years (*SD* 5.3) in cardiac and 76.0 years (*SD* 6) in non-cardiac patients.

**Intervention:**

The intervention arm consists of a multimodal, home-based tele-supervised prehabilitation programme over 2–4 weeks addressing deficits in physical fitness, nutrition, and preoperative anaemia.

**Primary outcome:**

Steps per day are assessed by an open-source algorithm from wrist-worn accelerometer data.

**Secondary outcome:**

Raw acceleration as overall Euclidean Norm Minus One (ENMO) is expressed in milligravitational units (m*g*).

**Results:**

Non-cardiac (*n* = 107) patients had more steps per day in the intervention group versus standard of care (4662 [2817; 6807] vs 3378 [1919; 4831], *p* = 0.042). Overall, ENMO was higher in the intervention group but not statistically significant. No significant differences in PA measures were observed between randomisation groups in cardiac (*n* = 60) patients. Cardiac patients had overall higher PA levels and were younger than non-cardiac. The difference remained after adjusting for age. Steps per day correlated strongly with overall ENMO.

**Conclusion:**

Simple preoperative PA recommendations effectively improve steps per day in high-risk non-cardiac surgery patients. To detect changes in PA in the high-risk surgical patient, steps per day can be used as an intuitive measure. To compare with other populations, overall ENMO is preferable.

**Trial registration number:**

ClinicalTrials.gov Identifier: NCT04461301.

**Supplementary Information:**

The online version contains supplementary material available at 10.1186/s13741-025-00554-4.

- What is already known on this topic?
  Physical activity (PA) levels in the high-risk surgical patient are low in the preoperative period. We designed a multimodal home-based prehabilitation intervention that includes recommendations with the aim to increase PA levels in the preoperative period. Whether home-based prehabilitation programmes are feasible and effective is unclear.
- What this study adds?
  This study demonstrates that a home-based tele-supervised prehabilitation programme increased steps per day in non-cardiac but not in cardiac patients in our hospital setting. This highlights the need to tailor prehabilitation according to surgical populations.
- How this study might affect research, practice, or policy?
  These findings underline the feasibility of using accelerometers to monitor PA in high-risk surgical populations. Based on our results, simple PA recommendations may be given to patients as part of a preoperative visit. In future studies, multiple measures of PA should be reported to increase robustness of results and allow comparisons to other populations.

## Introduction

Multimodal prehabilitation aims to optimise the health status of patients before surgery and therefore enhance postoperative recovery. Several trials have been published or are currently ongoing in different patient populations. While some trials indicate a potential reduction in postoperative complications by prehabilitation interventions, other results are less convincing (McIsaac et al. 2022).

Prehabilitation is resource-intensive and is reserved for patients suspected to be of high risk for postoperative complications. High-risk surgical patients are typically older, have reduced functional capacity, are generally fatigued, and are either over- or underweight (Whiteman et al. 2016). Given this phenotype, a supervised centre-based prehabilitation intervention is often not feasible, making home-based tele-supervised interventions a logical and scalable alternative. Several studies have introduced home-based programmes in recent years (Machado et al. 2024).

We developed a multimodal home-based tele-supervised prehabilitation programme that includes specific physical activity (PA) recommendations (Beilstein et al. 2023). We hypothesise that simple PA recommendations as a part of a multimodal prehabilitation intervention lead to an increase in steps per day in patients awaiting major surgery. To assess the effectiveness of such programs aiming at improving PA, objective assessment using accelerometers is recommended (VandeBunte et al. 2022). One metric that can be calculated from raw accelerometer data is steps per day. Steps per day are intuitive and widely used in research and clinical settings. Other metrics, such as overall raw acceleration described as Euclidean Norm Minus One (ENMO), may also be used to assess PA in the context of prehabilitation programs (Grimes et al. 2019).

The specific aims of this study are therefore as follows:

1. Assess whether the multimodal home-based tele-supervised prehabilitation intervention as utilised in the PREHABIL trial improves accelerometer-assessed steps per day in high-risk patients undergoing cardiac or non-cardiac surgery.
2. Assess how raw acceleration metrics (ENMO) correlate with steps per day in high-risk surgical patients in the preoperative period.

## Methods

### Recruitment

This is a sub-study of the PREHABIL trial, a multidisciplinary, two-arm parallel group randomised controlled superiority trial with blinded outcome assessment (Beilstein et al. 2023). High-risk patients undergoing major cardiac and non-cardiac surgery were randomised to either standard of care (preoperative management according to current clinical standards) or multimodal prehabilitation using a priori defined minimisation criteria in respect to surgery (cardiac vs non-cardiac) to allow subgroup analysis (Coart et al. 2023). Inclusion criteria were age ≥ 65 and a proven fitness deficit as measured by a cardiopulmonary exercise test (CPET). A fitness deficit was defined as either an oxygen uptake at the first ventilatory threshold (VO_2_@VT1) < 11 mL/min/kg or ventilatory efficiency (VE/VCO_2_ slope) ≥ 33.

After successfully recruiting 200 patients in the main study, we started to analyse the collected accelerometer data (defined as secondary outcome) according to surgical main categories. Data were collected between 02/2022 and 12/2024. Patients allocated to the intervention group received a tailored multimodal programme as described in the PREHABIL study protocol. In summary, the multimodal programme consists of a nutritional intervention focusing on energy and protein intake; an exercise component including respiratory, strength, and PA recommendations; and preoperative anaemia (defined as haemoglobin < 120 g/L in females and < 130 g/L in males) correction. The PA recommendations for elderly individuals were given to the patients according to the Swiss Federal Office of Health in either French or German (BAG, 2021). These recommendations advise patients to participate in aerobic exercise of moderate intensity of at least 150 min per week or in vigorous intensity for 75 min per week. Patients in the intervention group were followed up with weekly telephone calls to increase adherence to the intervention.

The PREHABIL trial is conducted in accordance with the good clinical practice recommendation of the Declaration of Helsinki and has been accepted by the responsible Ethics Committee of the Canton of Berne, Switzerland (Kantonale Ethikkomission Bern 2020–01690), and registered at ClinicalTrials.gov (NCT04461301). Written informed consent is obtained from all participants before entering the study.

### Sample size calculation

To detect a difference of 1000 steps per day and assuming a conservative standard deviation (1300) with a power of 80% and an alpha of 5%, we calculated a sample size of 28 patients for each group. The required sample size is 56 patients for cardiac and 56 patients for non-cardiac surgery patients, resulting in 112 patients with complete data.

### Physical activity monitoring

Triaxial accelerometers (Axivity AX-3, Axivity Ltd., Newcastle upon Tyne, UK) were worn continuously on the wrist for a minimum of 2 weeks before cardiac or non-cardiac surgery by all PREHABIL study participants. The dominant or nondominant wrist was used according to patient preferences with the aim to achieve high wear compliance. The AX-3 triaxial monitor records acceleration in the vertical, anteroposterior, and mediolateral axes. The data analysis framework described has been applied in previous research (Doherty et al. 2017; Eser et al. 2022). To configure the sensors, the open-source software Open Movement GUI V 1.0.0.45 was used (AX3 GUI 2024). The sampling frequency was set to 50 Hz and the dynamic range of sensors to ± 4 g to allow recording of data for up to 30 days. Recording started at 5 p.m. on the day the patient gave written informed consent to participate in the study. Patients were instructed to wear the sensor the entire preoperative period and to only take it off for bathing. They brought the sensor with them to the hospital when admitted for surgery, which was then collected by a member of the research team.

### Data processing

The raw accelerometer data were downloaded as a.cwa files using the Open Movement GUI software and stored in a designated research file directory. Files were then further processed using the research-driven open-source R package GGIR version 3.1–5 (van Hees & Migueles 2024). Criteria for data inclusion were defined as a minimum of 7 days with at least 12 h of daily wear time. Non-wear time was determined over a window size of 60 min with a 15-min sliding window. For the calculations of the movement component of the raw acceleration data, the default metric, which is the ENMO, was used. Basically, this metric describes the conversion of raw triaxial acceleration data into an omnidirectional measure of body acceleration (V. T. van Hees et al. 2013). Resulting ENMO values are expressed in gravity-based acceleration units (milligravitational units [m*g*]). Auto-calibration based on local gravity was conducted. Higher ENMO values indicate greater overall movement intensity, providing an objective measure of PA.

### Steps per day

Determination of steps was realised by a windows peak detection open-source algorithm. This algorithm is based on the design of Gu et al. (Gu et al. 2017) and implemented for use in combination with the GGIR package. The source code is available on GitHub (Patterson 2021) and we used the standard settings proposed by the authors.

### Statistical analysis

All analyses were performed with R version 4.2.3 (R Core Team 2024). We calculated descriptive statistics for all included participants by reporting variable means with standard deviations due to their primarily parametric distribution. The primary and secondary outcomes were tested for significance between groups using the Wilcoxon test, due to their primarily nonparametric distribution.

## Results

**Fig. 1:**
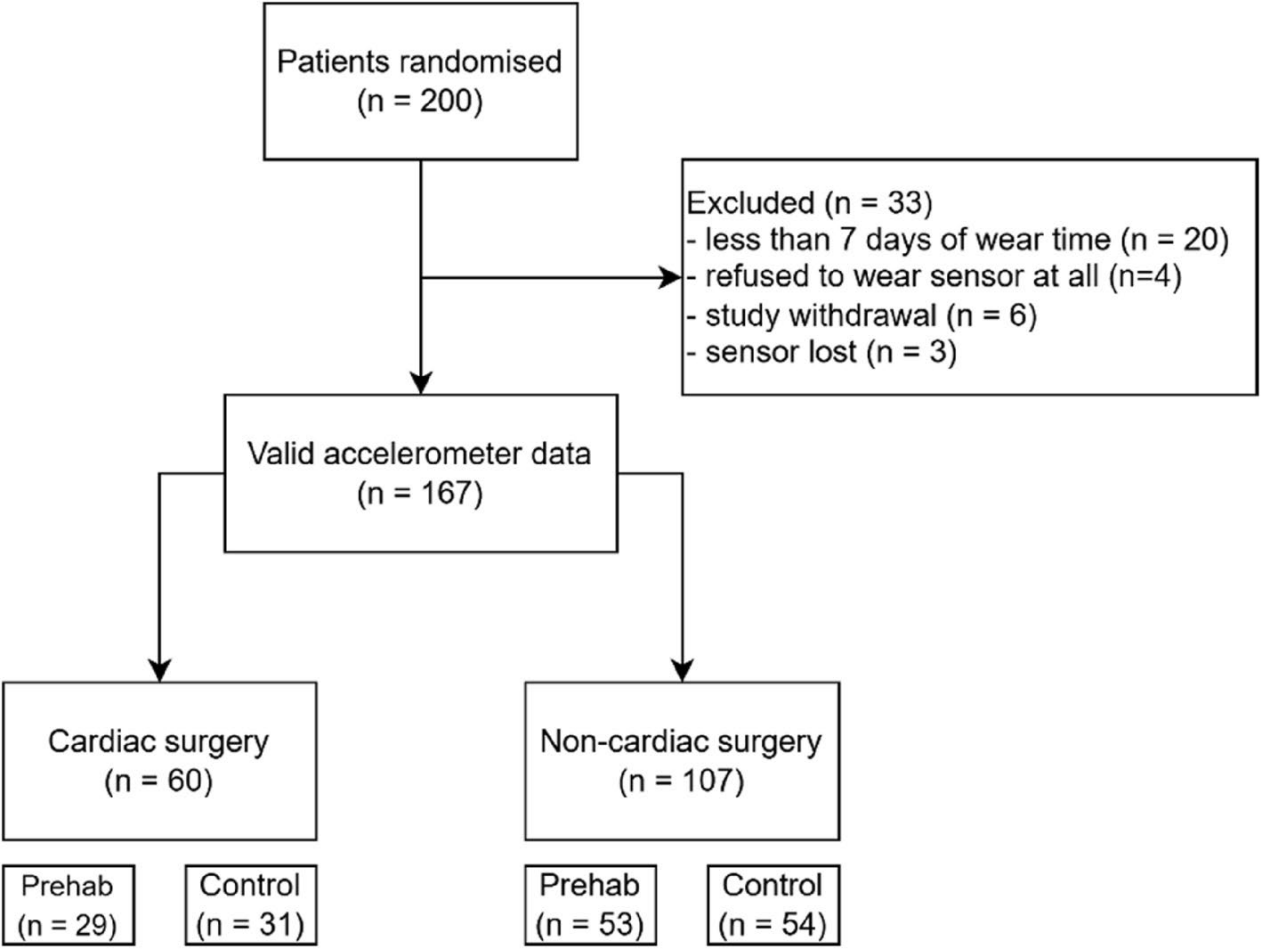
Patient flow of the study

Of 200 patients recruited for the PREHABIL trial, we could analyse 167 complete accelerometer files. Of the 33 patients excluded, 20 were excluded due to wear time of less than 7 days, 4 due to complete refusal to wear an accelerometer after giving consent, 6 due to study withdrawal, and 3 due to loss of the sensor (Fig. 1). Forty-four (24.7%) participants were listed for orthopaedic surgery, 25 (14.0%) for abdominal surgery, 21 (11.8%) for urological surgery, 21 (11.8%) for vascular surgery, 63 (35.4%) for cardiac surgery (bypass surgery and/or valve repair/replacement), and 4 (2.3%) for thoracic surgery.

Mean baseline values for the overall cohort were as follows: age 75.2 years (*SD* 5.85), female sex 25.7%, oxygen uptake at the first ventilatory threshold (VO_2_@VT_1_) 11.3 mL/min/kg (*SD* 2.83), oxygen uptake at peak exercise (VO_2_@peak) 14.7 mL/min/kg (*SD* 4.11), peak metabolic equivalents (METS) 4.19 (*SD* 1.17), haemoglobin value 131 g/L (*SD* 16.6), and number of valid accelerometer days was 20.7 (*SD* 5.4). Baseline characteristics according to main surgery classification (cardiac vs. non-cardiac) are available in Table 1, and detailed baseline characteristics according to randomisation groups are available in Supplemental Table S1.

**Table 1:** Baseline characteristics according to main surgery group (cardiac vs non-cardiac). Cardiac surgery patients are younger and have higher haemoglobin levels

	Cardiac	Non-cardiac
	* N = 60 *	* N = 107 *
Age (years)	73.8 (5.31)	76.0 (6.04)
Female	18 (30.0%)	25 (23.4%)
Surgery		
Cardiac	60 (100%)	0 (0.00%)
Orthopaedic	0 (0.00%)	44 (41.1%)
Thoracic	0 (0.00%)	4 (3.74%)
Urological	0 (0.00%)	20 (18.7%)
Vascular	0 (0.00%)	18 (16.8%)
Visceral	0 (0.00%)	21 (19.6%)
IHD yes	28 (47.5%)	38 (35.8%)
Diabetes: yes	20 (34.5%)	30 (28.3%)
COPD: yes	7 (12.1%)	13 (12.3%)
BMI	29.0 (5.62)	28.5 (5.43)
Valid sensor days	20.9 (5.26)	20.6 (5.50)
Power peak [watts]	73.6 (32.8)	72.4 (35.3)
VO_2_@VT_1_ [mL/min/kg]	11.7 (2.66)	11.1 (2.94)
VO_2_@peak [mL/min/kg]	14.9 (3.49)	14.6 (4.58)
VO_2_@peak predicted [%]	76.3 (15.4)	74.8 (19.4)
VE/VCO_2_ slope	38.9 (7.15)	39.5 (7.50)
RER peak	1.03 (0.10)	1.05 (0.12)
METs peak	4.25 (1.00)	4.18 (1.30)
METs DASI	5.62 (1.32)	5.16 (1.54)
Grip strength [kg]	28.4 (9.11)	26.8 (9.26)
Haemoglobin [g/L]	134 (14.0)	129 (17.7)

*IHD *Ischaemic heart disease, *COPD *Chronic obstructive pulmonary disease, *BMI *Body mass index, *VO*_*2*_*@VT1 *Oxygen uptake at the first ventilatory thresholds, *VO*_*2*_*@peak *Oxygen uptake at peak exercise, *MET *Metabolic equivalent, *DASI *Duke Activity Status Index, *RER *Respiratory exchange ratio

Self-reported adherence to the PA intervention at the first follow-up call (approximately 1 week after baseline visit) was 90%. The lowest self-reported adherence was found in patients undergoing orthopaedic surgery, where 38% of patients were not able to follow the recommendations due to movement-associated pain.

**Fig. 2:**
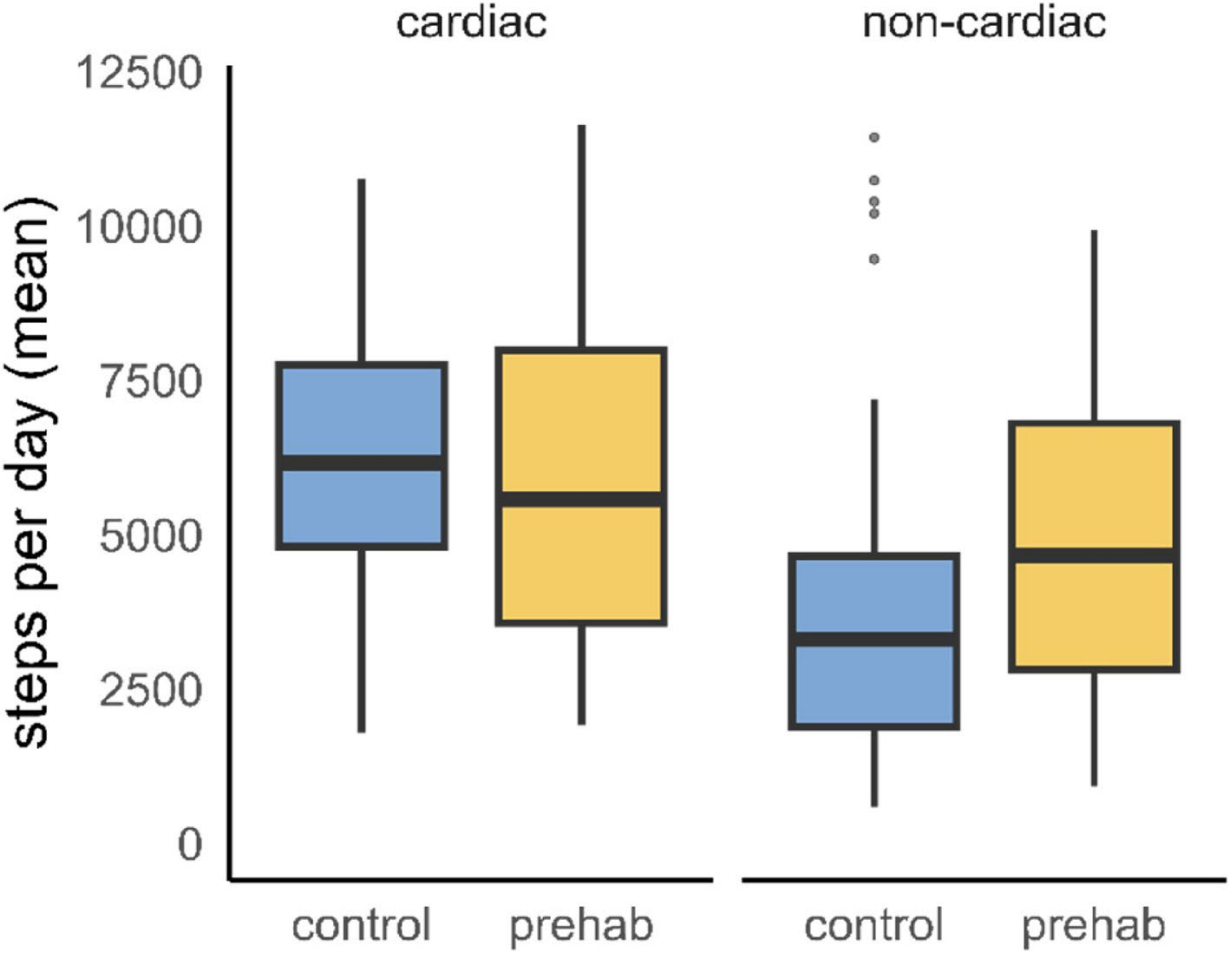
Primary outcome reported as steps per day. Patients undergoing non-cardiac surgery increased their daily step count through prehabilitation. This could not be observed in cardiac surgery patients. Cardiac surgery patients had overall higher activity levels compared to non-cardiac surgery patients

**Fig. 3:**
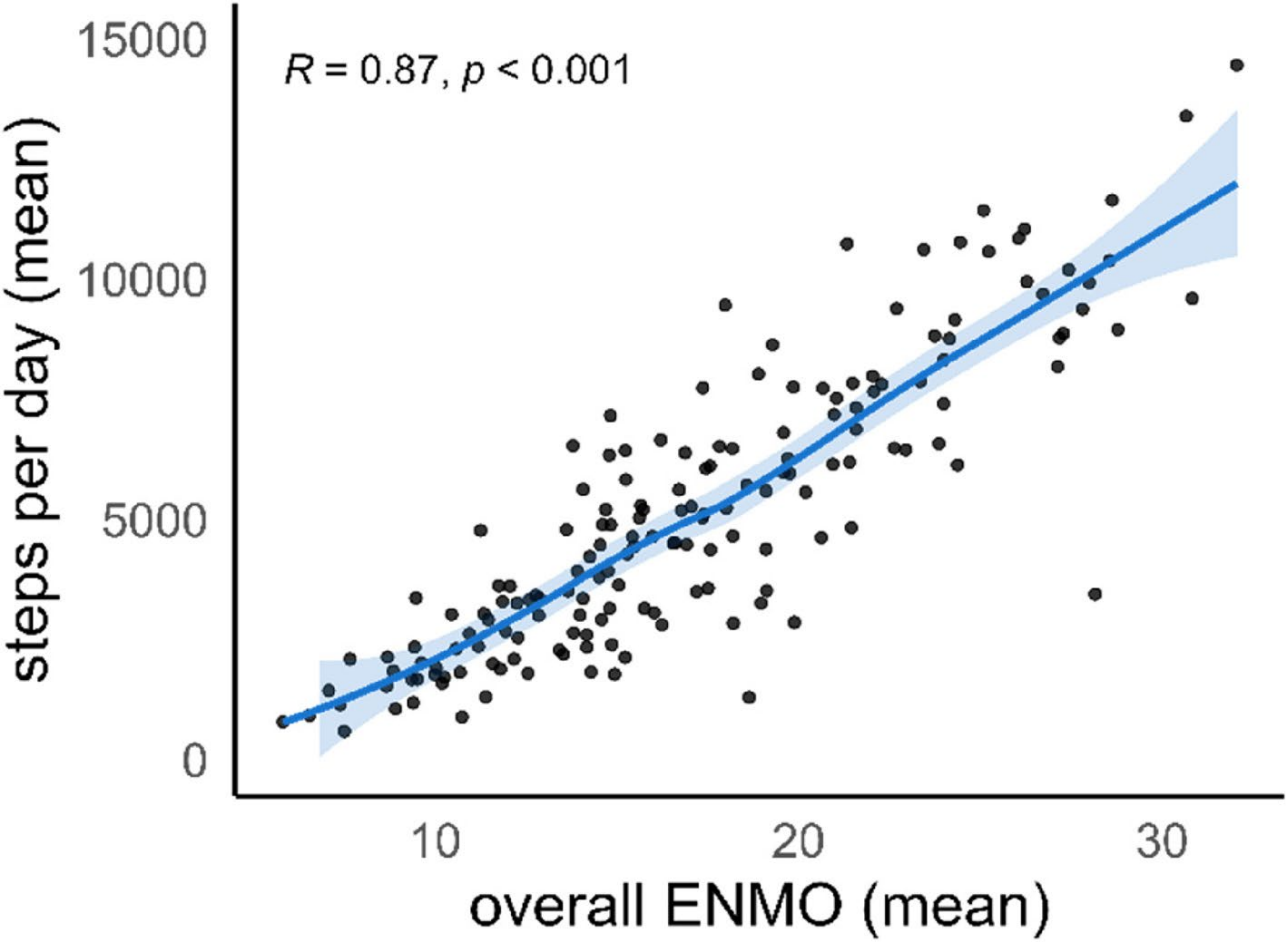
Steps per day versus overall Euclidean Norm Minus One (ENMO). Steps per day were correlated with overall ENMO expressed in milligravitational units (m*g*)

In patients undergoing cardiac surgery, the median value of steps per day was similar between randomisation groups (control 6190 [4842; 8042] vs intervention 5572 [3574; 7986], *p* = 0.631) (Fig. 2). Other measures of PA did also not differ between groups in cardiac surgery patients. In patients undergoing non-cardiac surgery, steps per day differed between randomisation groups (control 3378 [*IQR* 1919; 4831] vs intervention 4662 [*IQR* 2817; 6807], *p* = 0.042) (Fig. 2). Overall ENMO was 14.5 mg [*IQR* 11.4; 17.1] in the control and 16.9 mg [*IQR* 2.6; 19.3] in the intervention group (*p* = 0.094). PA outcomes other than steps per day are reported in the Supplemental Table S2. Irrespective of randomisation group, steps per day, time spent in light and moderate activity, and overall ENMO values were higher in cardiac vs non-cardiac surgery patients. Steps per day were directly correlated with overall ENMO values (Fig. 3).

## Discussion

We critically assessed objectively measured PA in patients participating in the PREHABIL trial, primarily expressed in steps per day. Accelerometer-based wrist-worn assessment of PA in the preoperative period was found feasible. Despite the logistic complexity of the preoperative period, we achieved valid accelerometer data from 167 of 200 (84%) recruited patients. We found a higher daily step count in patients undergoing non-cardiac surgery in the intervention group (multimodal prehabilitation) compared to standard of care (4662 versus 3378). Putting this finding in relation to existing evidence, in a cohort undergoing predominantly low-risk surgery (mean age 59 years), risk for postoperative complications was increased in patients taking less than 7500 steps/day in the preoperative period (Gehl et al. 2024). However, the minimally clinically important difference (MCID) in the preoperative setting for high-risk surgical patients is not yet established. For nonsurgical patient populations (mean age 69 years), a MCID of 350–1100 steps/day has been reported for the reduction of medical events (Teylan et al. 2018). From a pragmatic perspective, we consider the change we observed (+ 38% in the intervention group) as clinically relevant.

When looking at the overall ENMO in the non-cardiac population, we see a difference of 2.4 m*g* between the study groups. While the difference between the groups is not statistically significant, it is above the reported MCID of 1 m*g* (A. Rowlands et al. 2021).

We did not observe an effect of the intervention in patients undergoing cardiac surgery, who exhibited overall higher PA levels (in both groups) compared to non-cardiac. The higher PA levels may be explained by the fact that cardiac patients were younger and had higher haemoglobin levels, suggesting that this population is less frail compared to non-cardiac patients (Steinmeyer et al. 2020). The older age of the non-cardiac surgery patients did not account alone for the difference in PA levels between surgical categories. Even when adjusting for age, steps per day remained higher in cardiac patients, as reported in Supplemental Table S3. Patients awaiting cardiac surgery may have fulfilled inclusion criteria of low cardiorespiratory fitness predominantly due to cardio-circulatory limitations rather than muscular frailty, which could be more prevalent in the non-cardiac surgery population. A further possible explanation for the observed difference may lie in the context of the Swiss healthcare system. Most patients undergoing cardiac surgery had previously existing heart conditions, which resulted in referral to cardiac rehabilitation programs, which include PA recommendations and are reimbursed through compulsory basic health insurance.

While the validity of a different but comparable accelerometer (actiPAL™) to classify PA intensity in healthy young adults (mean age 24.8 years) has been reported (Lyden et al. 2017), the application of accelerometers in the high-risk surgical population has rarely been studied. The majority of the studies investigating preoperative populations included younger patients with less functional deficits (Wagnild et al. 2021). As part of an explorative analysis, we classified PA (light and moderate) according to published cut-points validated for elderly individuals (Migueles et al. 2021). Based on our analysis, classifying PA intensity using cut-points appears problematic as (1) validation for the cut-points in the high-risk population is lacking and (2) the definition of the bout duration criterion adds uncertainty to the analysis (Supplemental Fig. S1). Irrespective of the population, cut-points are generally a criticised analytical approach in the field of PA research (V. van Hees 2022; Hernández-Vicente et al. 2022; A. V. Rowlands et al. 2018).

We found that in our study population, overall ENMO was closely related to mean steps per day. ENMO is a relatively crude measurement that sums accelerations in all directions and averages them over each minute. Therefore, it does not rely on cut-points, algorithms, or time windows. It is an outcome that can be compared among different populations, largely independent of the software and hardware used for its assessment and calculation. An analysis using data from the UK Biobank study (*n* = 96,600) reported overall ENMO values of 23.9 m*g* for women and 22.9 m*g* for men aged 75–79 years (Doherty et al. 2017). We report (independent of randomisation group) values, which are 14.8 m*g* for non-cardiac and 19.7 m*g* for cardiac surgery patients. This demonstrates that our high-risk surgical population was significantly less active compared to the general population of the same age. Compared to the values reported by Grimes et al. (2019), where the study team assessed the effect of PA recommendations in non-cardiac surgery patients (mean age 79.9 years) pre- and post-intervention (14.3 m*g* [*IQR* 9.75; 22.04] vs. 20.91 m*g* [14.83: 27.53]), we report comparable ENMO levels in the control (14.5 m*g* [*IQR* 11.4; 17.1]) but not in the intervention group (16.9 m*g* [12.6; 19.3]). This difference may be explained by the study design (pre/post versus randomised).

Given the high feasibility and the manifold reasons to increase preoperative PA levels in the high-risk surgical population, we recommend providing wrist-worn accelerometers paired with simple recommendations to increase PA. Steps per day and overall ENMO can be used to track changes in PA. The metric that at present allows comparing absolute PA levels with other populations is overall ENMO due to its insensitivity to cut-points and/or algorithms.

### Limitations

This study has several limitations. First, even though the provided accelerometer does not provide any visual feedback, we expect that patients in the control group were likely motivated to increase PA due to knowing that they were monitored. This suggests that real “baseline” activity levels may be lower than reported in the standard of care group. Second, our primary outcome is steps per day, which was evaluated by an open-source algorithm not validated for the high-risk surgical population. Third, the reported steps per day primarily reflect walking activities. While this is the preferred mode of exercise for the majority of patients, other modes may not be detected (e.g. cycling). Fourth, patients wore the accelerometer on their wrist. This may be problematic for patients using a walker, as their hand remains stationary while walking, making step detection difficult. Fifth, PA recommendations were only one part of a multimodal prehabilitation programme. A standalone intervention focusing solely on PA may be more effective in doing so, and we cannot completely rule out that another component of the intervention affected PA in enrolled patients.

## Conclusion

Simple PA recommendations are effective in high-risk non-cardiac surgery patients in the frame of a tele-supervised multimodal prehabilitation programme to elevate the number of daily steps. Preoperative, objective PA assessment in the high-risk surgical patient can be achieved by measuring steps per day. To compare with other populations, overall ENMO is preferable and more robust.

## Supplementary Information

Additional file 1: Supplementary figure: Figure S1.Steps per day versus classification for “light” or “moderate” physical activity as proposed by published cut-points. Steps per day were best correlated with classification as “moderate PA”. Walking, which we assume the most intensive form of PA in the investigated frail population, would classify as “light PA”. The high amount of time spent in activity (either light and/or moderate), is rising questions regarding the face-validity of this analysis and demonstrates the challenges of cut-point PA classifications.Time spent in activity is largely depending on the bout threshold time that is applied (numerical values available in Table S2). Supplementary tables: Table S1. Detailed baseline characteristics for surgical category and randomisation group. IHD: ischaemic heart disease; COPD: chronic obstructive pulmonary disease; BMI: body mass index; VO_2_@VT1: oxygen uptake at the first ventilatory thresholds; VO_2_@peak: oxygen uptake at peak exercise; VO2@peak predicted : oxygen uptake at peak exercise of RER: respiratory exchange ratio. Table S2. Overall physical activity levels compared between cardiac and non-cardiac surgery patients. The primary outcome, steps per day, is reported as the median of all mean steps per day. Time spent in light and moderate PA were calculated according to published cut-points and are reported as unbouted, with 5 minute bouts and 10 minute bouts. Table S3. Steps per day adjusted for age between surgical and randomisation groups. The age difference does not explain the observed higher activity in cardiac patients. grp = randomisation group; surg = surgical group. glm = generalized linear model.

Additional file 2: Appendix 2. Reporting checklist for randomised trial

## Data Availability

The data that support the findings of this study are not openly available due to reasons of sensitivity and are available from the corresponding author upon reasonable request. Data are located in controlled access data storage at the University Hospital in Bern, Switzerland.
