# Supplementary material for: Effect of multimodal home-based prehabilitation on objectively measured physical activity in patients undergoing elective cardiac or non-cardiac major surgery: secondary outcomes from a randomised controlled trial
Source: Perioper Med (Lond). 2025 Jul 4;14:69. doi: 10.1186/s13741-025-00554-4 (PMC12228390; doi:10.1186/s13741-025-00554-4)
Supplement: Supplementary file 1 — Additional file 1: Supplementary figure: Figure S1.Steps per day versus classification for “light” or “moderate” physical activity as proposed by published cut-points. Steps per day were best correlated with classification as “moderate PA”. Walking, which we assume the most intensive form of PA in the investigated frail population, would classify as “light PA”. The high amount of time spent in activity (either light and/or moderate), is rising questions regarding the face-validity of this analysis and demonstrates the challenges of cut-point PA classifications.Time spent in activity is largely depending on the bout threshold time that is applied (numerical values available in Table S2). Supplementary tables: Table S1. Detailed baseline characteristics for surgical category and randomisation group. IHD: ischaemic heart disease; COPD: chronic obstructive pulmonary disease; BMI: body mass index; VO2@VT1: oxygen uptake at the first ventilatory thresholds; VO2@peak: oxygen uptake at peak exercise; VO2@peak predicted : oxygen uptake at peak exercise of RER: respiratory exchange ratio. Table S2. Overall physical activity levels compared between cardiac and non-cardiac surgery patients. The primary outcome, steps per day, is reported as the median of all mean steps per day. Time spent in light and moderate PA were calculated according to published cut-points and are reported as unbouted, with 5 minute bouts and 10 minute bouts. Table S3. Steps per day adjusted for age between surgical and randomisation groups. The age difference does not explain the observed higher activity in cardiac patients. grp = randomisation group; surg = surgical group. glm = generalized linear model. [file 13741_2025_554_MOESM1_ESM.docx]

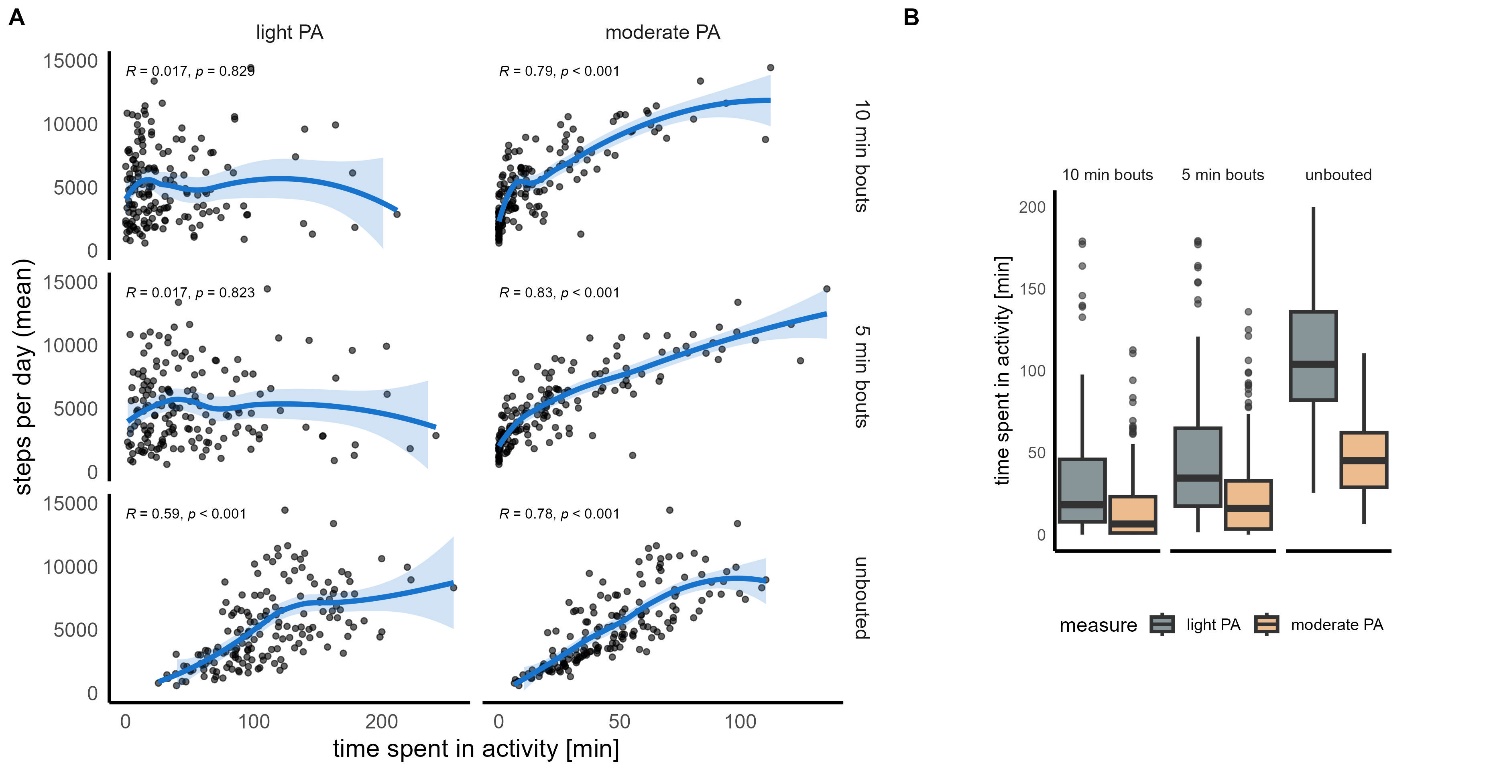


**Figure S1.** **(A)** Steps per day versus classification for “light” or “moderate” physical activity (PA) as proposed by published cut-points (Migueles et al., 2021). Steps per day were best correlated with classification as “moderate PA”. Walking, which we assume the most intensive form of PA in the investigated frail population, would classify as “light PA”. The high amount of time spent in activity (either light and/or moderate), is rising questions regarding the face-validity of this analysis and demonstrates the challenges of cut-point PA classifications.

**(B)** Time spent in activity is largely depending on the bout threshold time that is applied (numerical values available in **TableS2**).

**Table S1.** Detailed baseline characteristics for surgical category and randomisation group.

IHD: ischaemic heart disease; COPD: chronic obstructive pulmonary disease; BMI: body mass index; VO_2_@VT1: oxygen uptake at the first ventilatory thresholds; VO_2_@peak: oxygen uptake at peak exercise; VO2@peak predicted : oxygen uptake at peak exercise of RER: respiratory exchange ratio

|  | **cardiac** | | **non-cardiac** | |
| --- | --- | --- | --- | --- |
|  | **control** | **prehab** | **control** | **prehab** |
|  | ***N=31*** | ***N=29*** | ***N=54*** | ***N=53*** |
| Age [years] | 73.6 (5.47) | 74.1 (5.21) | 75.7 (6.16) | 76.3 (5.95) |
| Female: | 9 (29.0%) | 9 (31.0%) | 14 (25.9%) | 11 (21.6%) |
| IHD yes | 13 (43.3%) | 15 (51.7%) | 19 (35.8%) | 19 (35.8%) |
| Diabetes yes | 10 (34.5%) | 10 (34.5%) | 13 (24.5%) | 17 (32.1%) |
| COPD yes | 4 (13.8%) | 3 (10.3%) | 5 (9.43%) | 8 (15.1%) |
| BMI | 28.4 (5.98) | 29.6 (5.25) | 28.5 (5.68) | 28.5 (5.23) |
| Valid sensor days | 20.1 (5.29) | 21.7 (5.19) | 20.3 (5.77) | 21.0 (5.23) |
| Grip strength [kg] | 28.6 (9.13) | 28.3 (9.25) | 27.2 (9.73) | 26.3 (8.84) |
| Power peak [watts] | 78.4 (37.0) | 68.5 (27.4) | 74.3 (36.2) | 70.6 (34.6) |
| VO_2_@VT1 [mL/min/kg] | 11.6 (2.78) | 11.7 (2.58) | 11.5 (3.42) | 10.8 (2.33) |
| VO_2_@peak [mL/min/kg] | 15.1 (3.88) | 14.6 (3.08) | 15.0 (4.99) | 14.2 (4.12) |
| VO_2_@peak predicted [%] | 75.8 (16.1) | 76.9 (14.9) | 75.9 (19.1) | 73.5 (19.8) |
| VE/VCO2 slope | 37.9 (5.56) | 40.1 (8.48) | 40.4 (7.92) | 38.7 (7.00) |
| RER peak | 1.03 (0.10) | 1.02 (0.09) | 1.04 (0.14) | 1.05 (0.11) |
| Haemoglobin [g/L] | 133 (13.6) | 135 (14.5) | 131 (17.6) | 126 (17.6) |

**Table S2.** Overall (for both control and intervention group) physical activity (PA) levels compared between cardiac and non-cardiac surgery patients. The primary outcome, steps per day, is reported as the median of all mean steps per day. Time spent in light and moderate PA were calculated according to published cut-points (Migueles et al., 2021) and are reported as unbouted, with 5 minute bouts and 10 minute bouts.

|  | **cardiac** | | | **non-cardiac** | | |
| --- | --- | --- | --- | --- | --- | --- |
|  | **control** | **prehab** | **p** | **control** | **prehab** | **p** |
|  | ***N=31*** | ***N=29*** |  | ***N=54*** | ***N=53*** |  |
| Steps per day | 6190 [4842;8042] | 5572 [3574;7986] | 0.63 | 3378 [1919;4831] | 4662 4[2817;6807] | 0.04 |
| Overall ENMO [m*g*] | 19.9 [15.4;23.9] | 19.0 [15.7;23.9] | 0.83 | 14.5 [11.4;17.1] | 16.9 [12.6;19.3] | 0.09 |
| Light PA unbouted [min] | 115 [93.0;157] | 132 [103;171] | 0.23 | 94.9 [68.3;118] | 100 [85.4;127] | 0.15 |
| Light PA 5min [min] | 58.0 [27.3;90.4] | 33.1 [18.1;65.0] | 0.08 | 36.9 [18.0;62.0] | 30.9 [15.8;54.2] | 0.50 |
| Light PA 10min [min] | 37.7 [12.8;63.9] | 16.4 [5.74;44.0] | 0.06 | 17.9 [6.07;31.0] | 14.3 [7.65;34.7] | 0.99 |
| Moderate PA unbouted [min] | 51.3 [40.7;83.5] | 59.8 [40.8;72.2] | 0.98 | 35.5 [22.0;51.1] | 37.7 [29.6;61.7] | 0.06 |
| Moderate PA 5min [min] | 24.7 [8.16;41.0] | 18.7 [3.59;44.2] | 0.43 | 6.93 [2.20;20.8] | 18.5 [3.34;30.0] | 0.18 |
| Moderate PA 10min [min] | 12.4 [3.43;29.7] | 6.56 [2.18;32.4] | 0.54 | 2.80 [0.00;15.2] | 8.45 [1.28;21.1] | 0.10 |

**Table S3.** Steps per day adjusted for age between surgical (cardiac vs non-cardiac) and randomisation groups. The age difference does not explain the observed higher activity in cardiac patients.

grp = randomisation group (control or prehabilitation); surg = surgical group (cardiac or non-cardiac surgery)

**glm(mean_steps_per_day ~ grp * surg, data = data.complete, family = gaussian)**

| term | estimate | std.error | statistic | p.value | conf.low | conf.high |
| --- | --- | --- | --- | --- | --- | --- |
| (Intercept) | 6,608 | 479 | 13.793 | <0.001 | 5,669 | 7,547 |
| grpprehab | -540.7 | 700 | -0.773 | 0.441 | -1,912 | 831 |
| surgnon-cardiac | -2,546.3 | 605 | -4.206 | <0.001 | -3,733 | -1,360 |
| grpprehab:surgnon-cardiac | 1,356.4 | 871 | 1.557 | 0.121 | -351 | 3,064 |

**glm(mean_steps_per_day ~ grp * surg + age, data = data.complete, family = gaussian)**

| term | estimate | std.error | statistic | p.value | conf.low | conf.high |
| --- | --- | --- | --- | --- | --- | --- |
| (Intercept) | 13,791 | 2,656 | 5.192 | <0.001 | 8,586 | 18,997 |
| grpprehab | -472.6 | 683 | -0.692 | 0.490 | -1,811 | 866 |
| surgnon-cardiac | -2,333.8 | 595 | -3.920 | <0.001 | -3,501 | -1,167 |
| age | -97.8 | 36 | -2.747 | 0.007 | -168 | -28 |
| grpprehab:surgnon-cardiac | 1,345.4 | 852 | 1.579 | 0.116 | -325 | 3,015 |
